# Advances of exosomal miRNAs in the diagnosis and treatment of ovarian cancer

**DOI:** 10.1007/s12672-023-00674-7

**Published:** 2023-05-10

**Authors:** Jun Xiong, Fen Fu, Feng Yu, Xiaoju He

**Affiliations:** Department of Obstetrics and Gynecology, The Second Affiliated Hospital of Nanchang University, NanChang, JiangXi China

**Keywords:** Ovarian cancer, Exosomal miRNAs, Tumor microenvironment, Hallmark

## Abstract

Ovarian cancer is a tumor with the highest fatalities among female malignant tumors. This disease has no typical symptoms in its early stage, and most of the patients are in an advanced stage when being treated. The treatment effect is poor and it is easy to develop chemotherapy resistance. Therefore, it is particularly urgent to clarify the pathogenesis of ovarian cancer, explore its early diagnosis of biomarkers, and discover new treatment methods. As a carrier of intercellular information and genetic material transfer, exosomes are widely distributed in body fluids (e.g. blood and urine), which are regarded as latent tumor markers and take effects on tumor occurrence and invasion. Several articles have recently signified that exosomal miRNAs are widely implicated in the formation of the ovarian cancer tumor microenvironment, disease initiation and progression, and the generation of chemotherapy resistance. This article reviews the research on exosomal miRNAs in ovarian cancer.

## Introduction

Ovarian cancer is a primary malignancy in the female reproductive system, which is a tumor with the highest fatalities among gynecological malignancies and seriously threatens women’s health [1, 2]. The diagnosis and therapy of ovarian cancer have always been a clinical problem, because the early symptoms of the disease are inconspicuous, the treatment rate is low, and effective early diagnosis methods are lacking. When most patients are diagnosed, the disease has developed to the advanced stage [3]. At present, ovarian cancer is often treated by surgery, chemotherapy, and targeted therapy, but the five-year survival rate is only 47% [4, 5]. Traditional chemotherapy drugs lack targeting specificity for ovarian cancer cells, and have great side effects on patients and limited curative effect. However, targeted therapy is characterized by specific targets for tumor cells [6]. Therefore, in the era of precision medicine, it is urgently essential to uncover new markers for early diagnosing and effectively treating ovarian cancer [7, 8].

MicroRNAs are single-stranded RNAs with typical sequence lengths of 19–25 nucleotides, which are transcribed from the non-coding region of the genome [9]. At present, miRNAs have been found in multicellular eukaryotes such as animals, plants, and fungi [10]. However, there are differences in expression levels among different tissues, and there are significant differences in the expression level and distribution of miRNAs at different stages of body growth and development [9]. Although it only accounts for 1% of the entire human genome, at least 30% of gene expression is regulated by it [11]. It can target the specific sequence of 3 ‘-UTRs of mRNA and inhibit mRNA expression, thus playing an important role in various human tumor diseases [10, 12]. Because the expression profile of miRNA is more successful in classifying tumors that are close in composition than mRNA expression profiles [13], miRNA can be used as a biomarker for the diagnosis and prognosis of tumors [12, 14].

Exosomes are small vesicles ranging in diameter from 30 to 100 nm that are released from intracellular multicystic bodies [15], and originate from the endocytosis pathway [16]. As an important carrier of intercellular information and genetic material transfer [17], exosomes contain DNA, RNA, proteins, lipids, and other active substances, which can be used to monitor clinical status, treatment response, disease progression, and so on [18–21]. In recent years, exosomal miRNAs have attracted increasing attention [22]. As previously reported, exosomal miRNAs can function in the early diagnosis of malignancies, and they are tightly linked to the initiation and development of malignancies. Exosomal miRNAs secreted by tumor cells can promote the progression of malignant tumors by regulating the tumor microenvironment [23, 24]. This article reviews the research progress of exosomal miRNA ovarian cancer diagnosis and treatment.

### The overview of exosomal miRNAs

In 1981, Trams et al. identified by transmission electron microscopy a set of vesicle-like structures, and these structures had a smaller diameter in comparison to multivesicular bodies [25]. Subsequently, these membrane vesicles were successfully isolated from sheep reticulocytes through ultracentrifugation by Johnstone et al., and these vesicles were referred to as exosomes for the first time [26, 27]. Cell-secreted exosomes were initially thought to be cell damage-induced cellular waste, or cell homeostasis-caused by-products, and have no distinct impact on neighboring cells. Until recently, they are characterized as exocytosis-released extracellular vesicles after the fusion of multi-vesicular endosomes with the plasma membrane, which are widely distributed in body fluids such as blood and urine [20]. It is now believed that the formation of exosomes requires the following three steps: ①The formation of the initial endosomes at an early stage is achieved by the cell membrane invagination, and next, the bioactive substances start to gather in the early sorting endosomes; ②The early endosomes turn in late sorting endosomes under the control of the endocytic sorting complex and other relevant proteins essential for transport. Late sorting endosomes eventually form multivesicular bodies upon a second indentation; ③After the fusion of the multivesicular bodies and the cell membrane, the materials inside the cells, in the form of vesicles, are released to the outside [28], which are called exosomes.

MicroRNAs are endogenous non-coding RNAs, which lead to the target gene mRNA degradation or restrain their translation via specific mRNA complementary pairing of target genes [13, 29]. miRNAs modulate multiple pivotal biological functions, including differentiation, drug resistance proliferation, apoptosis, migration, as well as invasion via mediating gene expression [30–32]. Numerous studies showed they play essential roles in many biological processes, from normal development to disease processes [33, 34]. Therefore, miRNAs can be used as one of the indicators of tumor detection [35]. Because RNAs are easily degraded outside the cell [36], and miRNAs are not one-to-one corresponding to tumors [13], further research in this field is limited. It is worth noting that there are many kinds of exosomal RNAs, among which miRNAs are the most abundant [37, 38]. Packaging miRNAs into exosomes, they can not only avoid degradation, but also mediate cell-to-cell communication [39]. However, loading miRNAs into exosomes is a selective process, which does not simply reflect the maladjusted miRNA composition in parental cells [40]. It requires a special mechanism to sequence miRNAs in exosomes.

Diana et al. pointed out that miRNAs content in exosomes of KRAS-mutant colon cancer cells is different from KRAS-wild-type cancer cells: an enrichment of miR-10b was found in exosomes of wild-type KRAS cancer cells, whereas an enrichment of miR-100 was witnessed in KRAS-mutant cancer cells [41]. This outcome unveils that KRAS might mediate exosomal miRNA packaging. Some studies have found that AGO2, together with other RNA-binding proteins, also exerts function in regulating miRNAs loading into exosomes. During multivesicular body formation, AGO2 co-localizes with CD63 (an exosome protein) in the cytoplasm, and AGO2 phosphorylation alters exosomes release and miRNAs sorting and loading, causing miRNA uncoupling from the complex [42]. Sumoylated hnRNPA2B1 modulates the exosomal miRNA sorting via recognizing the miRNA 3’ end region GGAG and GGCU base sequences [43]. Other RNA-binding proteins (eg. neural sphingomyelinase 2 and Y-box protein 1) may also entrust specificity for miRNAs loading into exosomes [44, 45]. In short, how exosomes select specific miRNA loading may vary depending on tissue and cell metabolism [46]. The regulatory mechanism is still unclear and needs to be further explored in the future.

### Exosomal miRNAs function in ovarian cancer occurrence and development

**Fig. 1:**
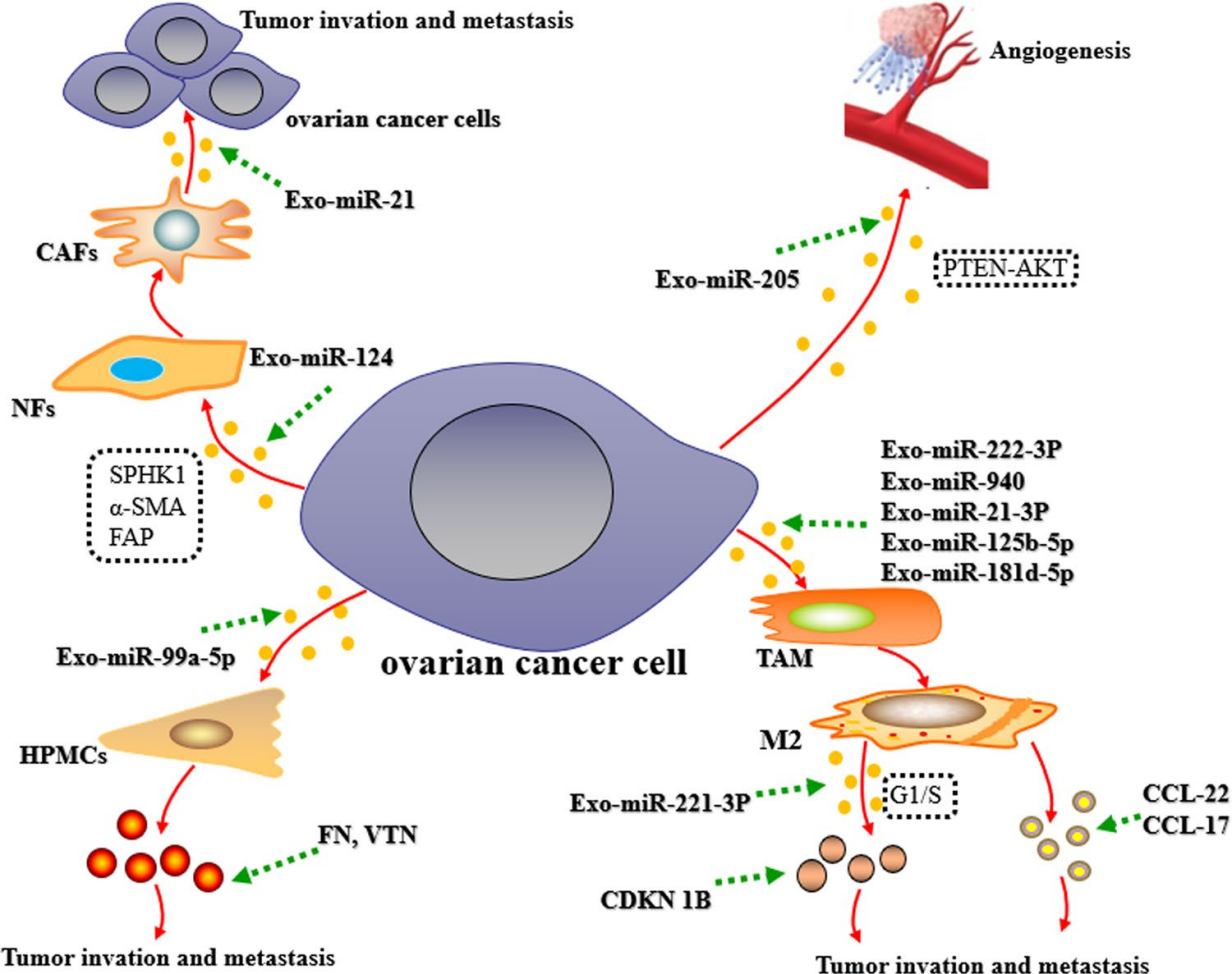
Exosomal miRNAs promote ovarian cancer initiation and development. Exosomal miR-124 from ovarian cancer targets SPHK1 and elevates FAP and α-SMA to differentiate NFs into CAFs and impede ovarian cancer cell apoptosis. Exosomal miR-205 from ovarian cancer cells induces angiogenesis to advance tumor growth and metastasis via the PTEN-AKT pathway. EOC cell-secreted exosomal miR-99a-5p boosts HPMC invasion through the upregulation of FN and VTN. Ovarian cancer cells-derived exosomal miRNAs (such as miR-125b-5p, miR-222-3p, miR-21-3p, miR-181d-5p, and miR-940) induces M2 polarization of macrophages. M2 macrophages are capable of advancing cancer progression via generating anti-inflammatory cytokines (such as chemokine 17 and chemokine 22) and exosomal miR-221-3p

The tumor microenvironment (TME) comprises cancer cells, endothelial cells, microvessels, immune cells, cancer-associated fibroblasts (CAFs), extracellular matrix (ECM), and biomolecules infiltrated [47]. The interaction between tumor cells and the microenvironment mediated by exosomal miRNAs is very important for the occurrence and development of tumors (Fig. 1).

As vital immune cells within the TME, tumor-associated macrophages (TAMs) are of significance in tumor cell proliferation and migration [48, 49]. TAMs are able to differentiate into M1 and M2 macrophages following different extracellular environments [50–52]. As reported, M1 macrophages hinder cancer progression via producing pro-inflammatory mediators (tumor necrosis factor-alpha and interleukin-1), while M2 macrophages advance cancer progression by means of anti-inflammatory cytokine secretion (chemokine 17, chemokine 22, and interleukin-10) [53–57]. Numerous studies have disclosed that exosomal miRNAs are capable of controlling the phenotypes of TAMs. Exosomal miR-222-3p and miR-940 from epithelial ovarian cancer (EOC) cells induces M2 macrophage polarization [58, 59]. In hypoxia, exosomal miR-125b-5p, miR-21-3p, miR-940, and miR-181d-5p from EOC cells can differentiate TAMs into M2 phenotypes, thereby enhancing tumor progression [60].

Exosomal miRNAs can modulate the TAM phenotypes and affect the disease progression via regulating the cell cycle and the reciprocity between immune cells and cancer cells [11]. For example, microarray analyses of exosomes demonstrated that 42 miRNAs had elevated expression in exosomes from M2 macrophages, and hsa-miR‐221‐3p was elevated 87.79 ± 9.0‐fold along all miRNAs. Furthermore, miR‐221‐3p suppressed cyclin‐dependent kinase inhibitor 1B (CDKN1B) directly. The study indicated that miR-221-3p contributed to EOC cell proliferation and G1/S transition [61]. Yoshimura et al. found that EOC cell-secreted exosomal miR-99a-5p boosts human peritoneal mesothelial cell (HPMC) invasion through the upregulation of fibronectin (FN) and vitronectin (VTN) [62]. Using next-generation sequencing technology, researchers at the University of Texas Anderson Cancer Center have identified higher miR-21 isomiRNA levels in tissue lysates and exosomes isolated from CAFs and cancer-associated adipocytes (CAAs) in contrast to those derived from ovarian cancer cells. Besides, functional studies revealed that miR-21 was transferred from CAFs or CAAs to ovarian cancer cells, where it suppressed cell apoptosis. Thus, the metastatic ovarian cancer cells’ malignant phenotype could be changed by exosomal miR-21 from neighboring stromal cells in the omental TME [63].

As an essential part of TME, ECM consists of protein and carbohydrates with functions such as support, connection, protection, water retention, as well as anti-stress [64]. ECM is able to support cell basic life activities, including migration, proliferation, as well as differentiation [65, 66]. Fibroblasts are the chief cell type and the primary source of ECM within the stroma [67, 68]. Normal fibroblasts (NFs) can suppress cancer-initiating and metastasis by means of ECM integrity, paracrine signaling, and direct cell-cell contact [69]. Nevertheless, exosomal miRNAs from tumors can induce some tumor-promoting signals, and induce the transformation of NFs into CAFs, which secrete factors such as extracellular matrix remodeling enzymes to alter the original physiological state of extracellular matrix and further modify the TME, thus supplying the optimal niche for cancer cells’ broad growth [69, 70]. For example, exosomal miR-124 from ovarian cancer targets sphingosine kinase 1 (SPHK1) and elevates fibroblast activating protein (FAP) and α-smooth muscle actin (α-SMA) to differentiate NFs into CAFs and controls CAF migratory and invasive capabilities, thus promoting tumor progression [71].

Tumor growth, metastasis, as well as invasion rely on angiogenesis, which provides the tumor cells with sufficient oxygen and nutrition [72, 73]. He et al. implicated that there was a high enrichment of miR-205 in cancer-adjacent endothelial cells, and restoration of miR-205 had positive relevance to high microvessel density in ovarian cancer patients. Exosomal miR-205 from ovarian cancer cells induces angiogenesis to advance tumor growth and metastasis via the PTEN-AKT pathway [74]. Therefore, exosomal miRNAs can affect ovarian cancer growth through multi-target, multi-level, and multi-pathway.

### Exosomal miRNAs as biomarkers of ovarian cancer

At present, the early diagnosis of tumors is limited by the late detection time and the cumbersome and expensive diagnostic process. Ideally, useful biomarkers should have specificity for specific tumor types and can be widely detected using non-invasive techniques in the pre-metastatic stage [75]. Despite the number of biomarker reports has steadily increased, few biomarkers approved by the US Food and Drug Administration have entered clinical practice., few FDA- approved biomarkers have reached the clinic to date [76, 77].

In contrast to traditional solid biopsies, which are impractical for screening or prognostic assays, tumor liquid biopsy is a new examination technique [19]. Because of its accuracy, non-invasive, dynamic, and repeatability, liquid biopsy has great clinical significance to detect and characterize the tumor [78]. The liquid biopsy consists of circulating tumor nucleic acids and tumor cells, free of cells or included in microvesicles, exosomes, and platelets [18, 79]. Therefore, many scholars detect exosomal miRNAs in blood, urine, along with other body fluids in order to find hallmarks for early diagnosis of ovarian cancer (Table 1).

Kazuya Maeda et al. collected sera samples from 58 patients with ovarian cancer before operation and found that early-stage ovarian cancer patients exhibited higher serum levels of exosomal miR-34a in contrast to those in an advanced stage, and the up-regulation of exosomal miR-34a was linked to lymph node metastasis. During the follow-up, it was found that patients in the recurrence-free group exhibited higher serum levels of exosomal miR-34a in contrast to those in the recurrence group [80]. These findings unveil that exosomal miR-34a could be utilized as a hallmark for early diagnosis of ovarian cancer, but also has important reference value for preoperative judgment of lymph node metastasis, the decision of surgical method, and evaluation of prognosis. He et al. found that there exhibited a specific elevation in serum exosomal miR-205 in ovarian cancer patients, and a dramatic elevation of miR-205 was witnessed in ovarian cancer patients with metastasis in contrast to those without metastasis [74].

MiRNA profiles were evaluated in exosomes from the plasma of 8 ovarian cystadenoma patients, 106 EOC patients, as well as 29 healthy women through the TaqMan real-time PCR‐based miRNA array cards consisting of 48 different miRNAs. Pan et al. demonstrated that miR‐21, miR‐320, miR‐100, and miR‐200b are enriched, whereas miR‐126, miR‐223, miR‐16, and miR‐93 are lowly expressed in exosomes from the plasma of EOC patients versus those in healthy women. Exosomal miR‐23a and miR‐92a exhibited lower levels in ovarian cystadenoma patients in contrast to those in EOC patients and healthy women, respectively [81]. Therefore, exosomal miRNAs can be used as a biomarker to judge whether patients have tumors and to differentiate benign from malignant tumors. Some studies have shown that miR-99a-5p harbored a reduced level in ovarian cancer-derived exosomes. Postoperative miR-99a-5p serum levels were reduced, uncovering that miR-99a-5p embodies tumor burden [62]. Thus, exosomal miR-99a-5p can be adopted as a potential hallmark for ovarian cancer diagnosis.

The expression levels of 3 members belonging to the miR-200 family were estimated in urine, plasma, as well as tumor tissues harvested from patients with ovarian cancer and benign ovarian tumor. No distinct difference was observed in the expression of target exosomal miRNAs in urine between the two groups. However, in the plasma and urine samples of ovarian cancer patients, the relative expression profiles of the three miRNAs were correlated, which supported the excretion of exosomal miRNAs into the urine [82]. Although urine exosomal miR-200 may not be a marker for ovarian cancer diagnosis, the analysis of miRNAs in the urine of ovarian cancer by Kun et al. disclosed that miR-10b level was the highest [83], so urine-derived exosomal miR-10b might be a specific biomarker for ovarian cancer diagnosis. Li et al. have stated that the peritoneal exosomes were isolated from 10 EOC patients with intraabdominal metastasis and 10 cancer-free participants. Through the next-generation sequencing, it was found that 249 miRNAs were highly expressed and 317 were lowly expressed in EOC samples versus participants without cancer samples. Following the Kaplan-Meier curve analysis, high miR-149-3p and miR-222-5p levels were linked to reduced 5-year and overall survival in EOC patients. High miR-1246 level had relevance with poor survival in EOC patients, but without statistical significance [84]. Therefore, peritoneal exosomal miR-149-3p, miR-222-5p, and miR-1246 may be new biomarkers for the diagnosis and evaluation of the prognosis of ovarian cancer patients.

**Table 1:** Expression of exosomal miRNAs in ovarian cancer patients

miRNAs	Origin	Expression	Significance	Prognosis	Refs.
miR-34a	Plasma	Up-regulated	A potential biomarker for improving the diagnostic efficiency of EOC	*	[80]
miR-205	Plasma	Up-regulated	Accelerated angiogenesis and tumour growth; a potential therapeutic target for EOC	*	[74]
miR-100	Plasma	Up-regulated	Indicating a selective packaging process and a potential occurrence of exosomes whose cargo also contains tumor suppressive potential	-	[81]
miR-21 miR-200b miR-320	Plasma	Up-regulated	Exosomal miR-21 displays oncogenic activity; the positive relationship of exosomal miR-200b with CA125 values; exosomal miR-320 has no impact on cell proliferation and apoptosis	miR-21: - miR-200b: * miR-320: -	[81]
miR-16 miR-93 miR-126 miR-223	Plasma	Down-regulated	Exosomal miR-16 is particularly associated with recurrence; exosomal miR-93, exosomal miR-126 and exosomal miR-223 act as tumor suppressors	miR-16: * miR-93: - miR-126: - miR-223: -	[81]
miR-23a miR-92a	Plasma	miR-23a: unchanged miR-92a: down-regulated	Exosomal miR-23a and miR‐92a can be used as potential biomarkers for differentiating benign from malignant tumors	-	[81]
miR-99a-5p	Plasma	Up-regulated	Promotes cell invasion by affecting HPMCs at least in partly through fibronectin and vitronectin upregulation	-	[62]
miR-200a miR-200b miR-200c	Urine	Unchanged	In the plasma and urine samples of patients with ovarian cancer, the relative expression profiles of the three miRNAs were correlated, which supported the excretion of exosomal miRNAs into the urine	-	[82]
miR-10b	Urine	Up-regulated	Exosomal miR-10b was the most highly expressed microRNA in all the samples	-	[83]
miR-149-3p miR-222-5p miR-1246	Ascites	Up-regulated	Exosomal miR-149-3p and exosomal miR-222-5p were significantly enriched in 19 pathways, including the antigen processing and presentation pathway	*	[84]

* refers to prognostic importance of exosomal miRNAs

### Exosomal miRNAs as therapeutic tools

In cancer treatment, exosomes have many unique characteristics and can be used for research in drug delivery nanocarriers, cancer vaccines, and antigen carriers [85]. Compared with commercial liposomes and polymer nanoparticles, exosomes have many advantages as drug delivery carriers due to their biocompatibility, low immunogenicity, and innate ability to interact with target cells [86]. Exosomes mediate intercellular communication and express various kinds of adhesion proteins on the surface, which can accelerate membrane interaction and fusion. Through exosomes, donor cells can transfer foreign substances including miRNAs to recipient cells. Therefore, the construction of artificial exosomes as advanced targeted drugs for cancer treatment has become a hot spot [87–89].

Exosomes can also serve as a new type of gene therapy vector, used for the treatment of various genetic diseases and cancers by transporting gene substances such as miRNA and DNA [86]. For instance, Nikolas et al. loaded pro-apoptotic miR-744 and miR-493 into exosomes through genetic engineering and found that these miRNAs were not only absorbed by ovarian cancer cells, but also promoted cancer cell apoptosis by down-regulating related target genes [90]. Hu et al. found that exosomes from macrophages that had been treated with TNF-like weak inducer of apoptosis can inhibit ovarian cancer cell metastasis via expressing miR-7 [91]. Wei et al. pointed out that miR-199b-3p suppressed ovarian cancer progression by mediating zinc finger E-box binding homeobox 1 [92]. According to these reports, it can be found that using exosomes as a tool to load miRNAs in ovarian cancer therapy is a very promising direction.

**Fig. 2:**
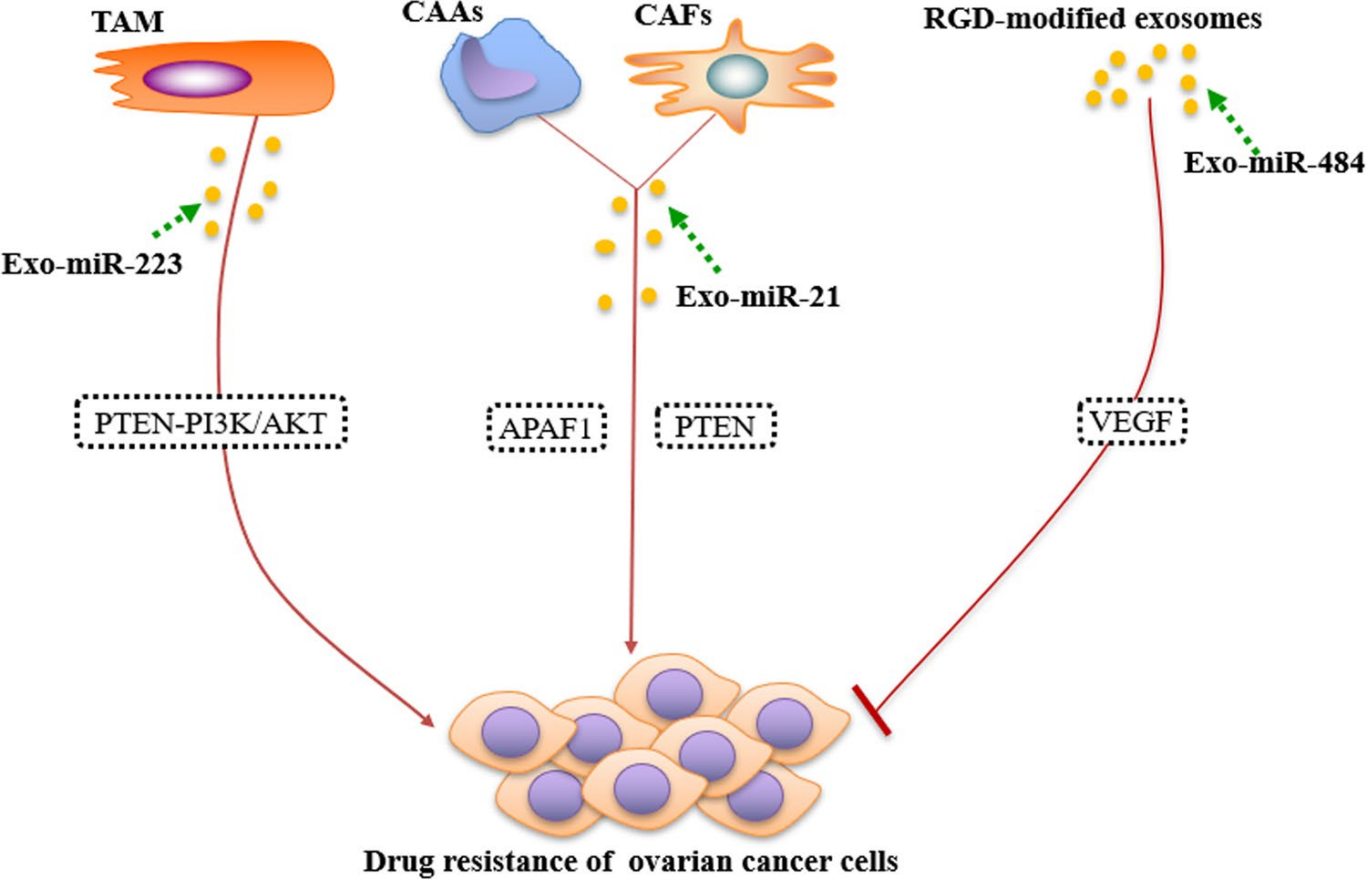
Exosomal miRNAs involved in chemotherapy resistance of ovarian cancer. Macrophage-secreted exosomal miR-223 advanced the drug resistance of EOC cells via the PTEN-PI3K/AKT pathway both in animal and cellular assays. CAAs and CAFs-derived exosomal miR-21 is able to induce drug resistance of ovarian cancer cells through binding APAF1 and down-regulating PTEN. Targeted delivery of miR-484 via RGD-modified exosomes could inhibit VEGF significantly and sensitizes the cancer to chemotherapy

Chemotherapy is a chief method for treating ovarian cancer, but most patients will experience multiple chemotherapies due to recurrence. After each chemotherapy, the tumor-free survival period of patients gradually shortens and changes from platinum sensitivity to platinum resistance. Therefore, chemotherapy resistance is a thorny problem in ovarian cancer therapy [93, 94]. The role of exosomal miRNAs in chemotherapy resistance in patients with ovarian cancer has attracted widespread attention (Fig. 2). For example, Zhu et al. found that macrophages-derived exosomal miR-223 boosted the drug resistance of EOC cells via the PTEN-PI3K/AKT pathway both in animal and cellular assays, and circulating exosomal miR-223 levels had relevance with the recurrence of EOC [95]. Exosomal miR-21 secreted from adjacent stromal cells in omental TME can induce ovarian cancer cell drug resistance via binding apoptotic protease activating factor-1 (APAF1) and down-regulating phosphatase and tensin homolog (PTEN) [11, 63]. Zhao et al. found that targeted delivery of miR-484 via RGD-modified exosomes improves the vascular normalization, which in turn sensitizes the ovarian cancer to chemotherapy, and prolongs the survival time of tumor-bearing mice after chemotherapy [96]. In summary, exosomal miRNAs are expected to open a novel door for the therapy of drug-resistant ovarian cancer patients.

## Conclusions

To sum up, ovarian cancer is a severe threat to women’s health, and the main problems we face are still the difficulty of early diagnosis, unclear pathogenesis, and severe chemotherapy resistance. Exosomal miRNAs have the significant characteristics of biomarkers and are easy to obtain, so they have unparalleled advantages in the early diagnosis of the disease, and also provide a new perspective for us to understand the mechanism of ovarian cancer. The pathogenesis and drug resistance mechanism of ovarian cancer are multifactorial. Most of the studies of exosomal miRNAs are still in vitro or animal experimental stages, and there are few clinical application studies. Therefore, the relationship between exosomal miRNAs and ovarian cancer initiation and progression and the application of exosomal miRNAs in tumor therapy still need to be further explored.

## Data Availability

All data we used in this work can be found in the references.

## Abbreviations

TME: Tumor microenvironment
CAFs: Cancer-associated fibroblasts
ECM: Extracellular matrix
TAMs: Tumor-associated macrophages
EOC: Epithelial ovarian cancer
CDKN1B: Cyclin‐dependent kinase inhibitor 1B
HPMCs: Human peritoneal mesothelial cells
FN: Fibronectin
VTN: Vitronectin
CAAs: Cancer-associated adipocytes
NFs: Normal fibroblasts
SPHK1: Sphingosine kinase 1
α-SMA: α-smooth muscle actin
FAP: Fibroblast activating protein
APAF1: Apoptotic protease activating factor-1
PTEN: Phosphatase and tensin homolog
SOX9: SRY-box 9
